# Silodosin versus tamsulosin for medical expulsive treatment of ureteral stones: A systematic review and meta-analysis

**DOI:** 10.1371/journal.pone.0203035

**Published:** 2018-08-28

**Authors:** Yuan-Pin Hsu, Chin-Wang Hsu, Chyi-Huey Bai, Sheng-Wei Cheng, Kuan-Chou Chen, Chiehfeng Chen

**Affiliations:** 1 Emergency Department, Wan Fang Hospital, Taipei Medical University, Taipei, Taiwan; 2 Graduate Institute of Clinical Medicine, College of Medicine, Taipei Medical University, Taipei, Taiwan; 3 Department of Emergency, School of Medicine, College of Medicine, Taipei Medical University, Taipei, Taiwan; 4 Department of Public Health, School of Medicine, College of Medicine, Taipei Medical University, Taipei, Taiwan; 5 Division of Gastroenterology, Department of Internal Medicine, Wan Fang Hospital, Taipei Medical University, Taipei, Taiwan; 6 Department of Urology, Taipei Medical University Shuang-Ho Hospital, Taipei, Taiwan; 7 Cochrane Taiwan, Taipei Medical University, Taipei, Taiwan; 8 Division of Plastic Surgery, Department of Surgery, Wan Fang Hospital, Taipei Medical University, Taipei, Taiwan; 9 Evidence-based Medicine Center, Wan Fang Hospital, Taipei Medical University, Taipei, Taiwan; University of Mississippi Medical Center, UNITED STATES

## Abstract

Silodosin, a recently introduced selective α-blocker, has a much higher selectivity for the α-1A receptor. The efficacy and safety of silodosin compared to tamsulosin in medical expulsive therapy (MET) are controversial. The objective of this study was to assess the efficacy and safety of silodosin compared to tamsulosin for treating ureteral stones <10 mm in diameter. We systematically searched the PubMed, EMBASE, Cochrane library, and Scopus databases from their inception to May 2018. We included randomized controlled studies (RCTs) and observational studies, which investigated stone expulsion rates using silodosin compared to tamsulosin. Data were synthesized using a random-effects model. Sixteen studies with 1824 patients were eligible for inclusion. Silodosin achieved significantly higher expulsion rates than tamsulosin (pooled risk difference (RD): 0.13, 95% confidence interval (CI): 0.09 to 0.18, GRADE: high). A subgroup analyses showed that silodosin has a significantly higher expulsion rate on stone sizes of 5–10 mm than tamsulosin (pooled RD: 0.14, 95% CI: 0.06 to 0.22, I^2^ = 0%). The superior effect was not observed on stone sizes <5 mm. A multivariate regression showed that the RD was negatively associated with the control expulsion rate after adjusting for age and gender (coefficient -0.658, *p* = 0.01). A sensitivity analysis showed that our findings were robust. Patients receiving silodosin also probably had a significantly shorter expulsion time (pooled mean difference (MD): -2.55 days, 95% CI: -4.06 to -1.04, I^2^ = 85%, GRADE: moderate) and may have fewer pain episodes (pooled MD: -0.3, 95% CI: -0.51 to -0.09, GRADE: low) but a higher incidence of retrograde ejaculation by 5% compared to those receiving tamsulosin. In conclusion, compared to tamsulosin, silodosin provided significantly better stone passage for patients with ureteral stones (particularly for sizes of 5~10 mm), shorter expulsion times, and fewer pain episodes but caused a higher incidence of retrograde ejaculation.

## Introduction

Ureteral stones are a common problem in primary care practice [1], with observed incidences of 3%~18% in various geographical locations [2]. Patients with ureteral stones have a reduced quality of life because ureteral stones are one of the most painful urologic disorders [3]. Furthermore, with an increasing prevalence in the US, the economic burden is also growing [4].

The management of ureteral stones includes watchful waiting for spontaneous passage, medical expulsive treatment (MET), extracorporeal shock wave lithotripsy, ureterorenoscopic lithotripsy, open ureterolithotomy and laparoscopic ureterolithotomy. Except watchful waiting and MET, the other interventions have higher healthcare expenditures and are relatively invasive, so the MET is preferred by patients, as it might facilitate the spontaneous expulsion of ureteral stones [5]. Several pharmacological agents are used in MET, including α-blockers, calcium channel antagonists, phosphodiesterase inhibitors, and corticosteroids. These have been demonstrated to facilitate ureteral stone passage. Of these interventions, α-blockers have the highest ranking, and the most commonly used α-blocker is tamsulosin [6].

Silodosin, a recently introduced selective α-blocker, has a much higher selectivity for the α-1A receptor. Recent meta-analyses that included few randomized controlled trials (RCTs) demonstrated that silodosin is superior to tamsulosin for the expulsion of ureteral stones [7–11]. However, those studies had important limitations, including low numbers and small sample sizes of the RCTs, which made it difficult to perform a subgroup analysis of stone sizes, which affects the probability of spontaneous passage. With an increasing number of published studies that investigated the efficacy and safety of silodosin versus tamsulosin on the expulsion of ureteral stones, we conducted a comprehensive systematic review with a meta-analysis and trial sequential analysis (TSA) to evaluate the efficacy and safety of silodosin versus tamsulosin in MET for ureteral stones.

## Materials and methods

We followed the preferred reporting items for systematic review and meta-analyses (PRISMA) guidelines (S1 Checklist) for this meta-analysis [12] and registered it at PROSPERO (PROSPERO ID: CRD42018094025).

### Search strategy and study selection

A literature search was performed in the PubMed, EMBASE, Cochrane library and Scopus databases using eligibility criteria with the following search terms: silodosin, tamsulosin, medical expulsive therapy, ureteral stone, and urolithiasis (S1 Table). We also manually searched the references of recently published relevant articles. The last literature search was performed in May 2018.

### Inclusion and exclusion criteria

All published human RCTs, prospective cohort studies, and retrospective cohort studies comparing silodosin with tamsulosin to manage ureteral stones of sizes <10 mm were considered for inclusion. Case reports, case series, and studies that reported on patients who received SWL were excluded. In addition, we identified other studies using the reference sections of relevant papers and by corresponding with subject experts. Finally, unpublished studies were collected from the ClinicalTrials.gov registry (http://clinicaltrials.gov/). No language restrictions were applied.

### Outcomes of interest

Our primary outcome of interest was the expulsion rate. The stone expulsion rate was defined as the rate of patients with spontaneous stone expulsion without an intervention during the study period. Secondary outcomes were the expulsion time, number of pain episodes, requirements for analgesics, and adverse events associated with silodosin versus tamsulosin.

### Data extraction and management

Baseline and outcome data were independently abstracted by two reviewers (CC and YPH), and the study designs, study population characteristics, inclusion and exclusion criteria, method of intervention, complications, and post-treatment parameters were extracted. Decisions individually recorded by the reviewers were compared, and disagreements were resolved by a third reviewer (CHB). The authors of the studies were contacted for additional information if required.

### Assessment of risk of bias in the included studies

Two reviewers (CC and YPH) independently assessed the methodological quality. For RCTs, we used the risk of bias method recommended by the Cochrane Collaboration [13], which includes domains of randomization, allocation and concealment, blinding of participants and personnel, blinding of outcome assessors, incomplete outcome data, reporting bias and other biases. For observational studies, we used the Newcastle-Ottawa scale tool [14], which has three domains based on selection of the cohort, comparability of the groups, and quality of the outcomes. The results were summarized in a risk of bias table. In addition, any disagreements on the quality assessment were resolved through comprehensive discussions.

### Statistical analysis

#### Measures of the treatment effect

We analyzed outcomes as continuous or dichotomous data using standard statistical techniques with a random-effects model up to the end of follow-up. For continuous outcomes, we used the mean difference (MD) and 95% confidence interval (CI). For dichotomous outcomes, we calculated the risk difference (RD) with the 95% CI. If some of the continuous data were given on different scales, we produced the results as the standardized mean difference (SMD) and 95% CI.

#### Assessment of heterogeneity

We used the I^2^ statistic and χ^2^ test to measure heterogeneity among studies in each analysis. Heterogeneity was categorized as low (<30%), moderate (30%~60%), or high (>60%) based on the I^2^ values [15]. If we identified substantial heterogeneity, we reported this and explored possible causes by performing prespecified subgroup analyses (stone sizes (>5 vs. <5 mm), stone location (only distal ureter stones (DUSs) vs. not only DUSs), follow-up times (1, 2, 3, and 4 weeks), and study design (RCT vs. observational study). Additionally, a sensitivity analysis was performed to better understand the sources of statistical heterogeneity between studies, as well as test the robustness of our findings based on RCTs excluded because of only having an abstract, excluded because of high or unclear risk in each domain of the risk of bias, excluded because of unclear information about ages or stone sizes, and excluded because of unclear information on the measurement of stone passage and hydration. Outcome measures were cross-validated using the relative ratio (RR) and odds ratio (OR). Furthermore, we applied a meta-regression to assess relationships of age, gender, stone sizes, laterality of the stone location, and control expulsion rate (defined as the expulsion rate in the tamsulosin group) with the primary outcome using Comprehensive Meta-Analysis software (vers. 3.3.070, Biostat, Inc., Englewood, NJ, USA)

#### Trial sequential analysis

A TSA was performed to reduce the risk of random errors, increase the robustness of the meta-analyses, and determine whether the current sample size was sufficient [16, 17]. TSA monitoring boundaries for the meta-analysis and the required information size (RIS) were quantified and adjusted CIs were calculated. The RIS indicates a target sample size considering the heterogeneity of the data. The risk of a type 1 error was set to 5% with a power of 90%. A relative risk reduction of 15% for the expulsion rate was considered clinically significant [18]. If the cumulative z-curve crosses the trial sequential monitoring boundary, a sufficient level of evidence has been reached and no further trials are needed. If the z-curve does not cross the boundary and the required information size has not been reached, there is insufficient evidence to reach a conclusion. The TSA program vers. 0.9 beta (www.ctu.dk/tsa) was used for the TSAs.

#### Assessment of reporting biases

Publication bias was assessed by detecting asymmetry in funnel plots if at least 10 studies were included. We used Egger’s test to examine possible small study effects [19].

#### Grading the quality of evidence

The quality of the evidence for each outcome was assessed by two independent team members (CC and YPH), using the grading of recommendations assessment, development, and evaluation (GRADE) methodology [20]. The quality of evidence was classified as high, moderate, low, or very low based on judgments of the risk of bias, inconsistency, imprecision, indirectness, and publication bias [20]. We resolved discrepancies by consensus, and if needed, with arbitration by a third team member (CHB).

## Results

### Results of the search

**Fig 1:**
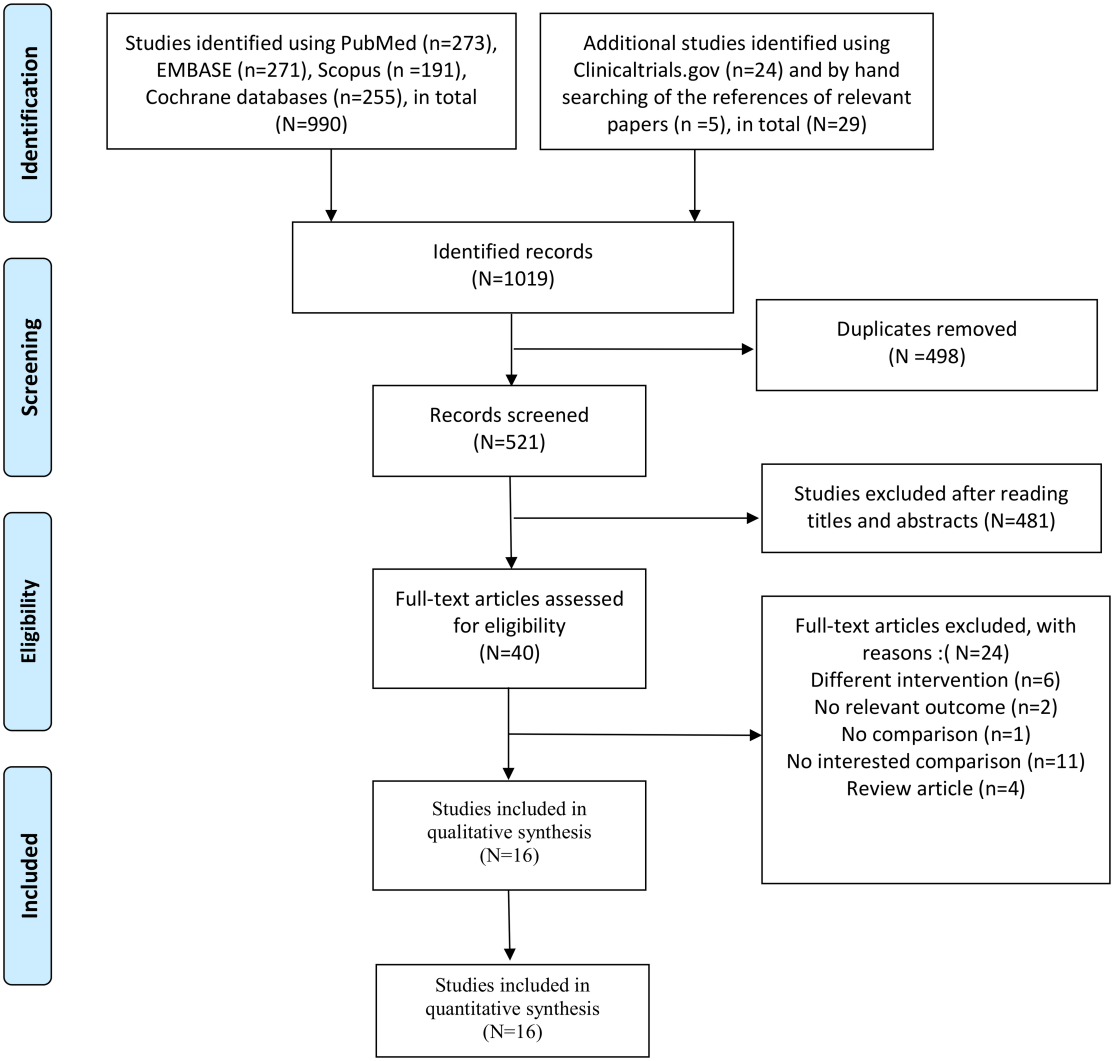
Flow diagram of the search process and search results.

Fig 1 shows the screening and selection processes of the study. Our initial search yielded 990 studies from PubMed, EMBASE, Cochrane Library and Scopus, 5 studies from hand searching of the reference sections of relevant papers and 24 studies from Clinicaltrials.gov. After duplicates were removed, 521 studies remained, of which 481 articles were deemed ineligible after screening the titles and abstracts. Full-text articles were excluded with different interventions (*n* = 6), no relevant outcome measure (*n* = 2), no comparison (*n* = 1), and no comparison of interest (*n* = 11), as well as review articles (*n* = 4). Sixteen studies were included for qualitative and quantitative synthesis.

### Study characteristics

A complete overview of the characteristics of the included studies is given in Table 1 and S2 Table. Thirteen studies [18, 21–32] were RCTs, and three [33–35] were observational studies. Two RCTs [25, 30] provided only an abstract. The study sample sizes ranged from 59~315, with 1824 total. These studies were conducted in India [21, 22, 27–32], Italy [18, 23, 34], Romania [26], Egypt [24, 25], and Turkey [33, 35]. The average age of the participants ranged from 32~53.5 years. The average stone sizes ranged from 4.2~7.4 mm. There were no significant differences between respective groups regarding sex, age, or stone size. In terms of stone location, 13 studies [18, 22–25, 28–35] included patients with DUSs, and three studies [21, 26, 27] not only focused on DUSs but also on middle ureteral stones or any location of ureteral stones. Most trials used kidney, ureter, bladder (KUB), ultrasound, or computed tomography (CT) for diagnosing ureteral stones. To measure stone passage, most studies used a combination of the patient’s statement, KUB, US, and/or CT, and one study [32] used only the patient’s statement. All studies used 8 mg silodosin per day compared to 0.4 mg tamsulosin per day. For co-medication, there were variations in the doses of analgesics and the hydration status. The dropout rate was low across the studies, except for one study [28]. The time of follow-up ranged from 2~4 weeks.

**Table 1:** Characteristics of the included trials.

Study	Design/Setting	Sample size:	Age: mean (SD), years	Stone size: mean (SD), mm	Inclusion criteria	Diagnosis/ measurement of stone	Measurement of stone passage	Co-medication	Intervention and placebo method	Dropout *n* (S/P)	Follow-up
Gupta [27] 2013	RCT/India, 1 center	S: *n* = 50 T: *n* = 50	S: NA T: NA	S: 6.6 (1.8) T: 7.0 (2.3)	Size: <10 mm Location: middle or lower	KUB, CT/ greatest dimension	Patient’s statement, KUB, US	Diclofenac 100 mg prn Hydration: NA	S: 8 mg qd T: 0.4 mg qd	0/0	4 weeks
Rathi [30] 2014	RCT/India, 1 center	S: *n* = 29 T: *n* = 30	S: NA T: NA	S: NA T: NA	Size: <10 mm Location: distal	NA	NA	Diclofenac regular for 1 week and then prn Hydration: NA	S: 8 mg qd T: 0.4 mg qd	NA	4 weeks
Imperatore [34] 2014	Retrospective/Italy, 1 center	S: *n* = 50 T: *n* = 50	S: 50.1 (NA) T: 53.5 (NA)	S: 6.5 (NA) T: 6.7 (NA)	Size: <10 mm Location: distal	Radiopaque/ large dimension	Filter, KUB	Diclofenac 75 mg prn Hydration: 2 L/day at least	S: 8 mg qd T: 0.4 mg qd	0/0	4 weeks
Kumar [18] 2015	RCT/Italy, 1 center	S: *n* = 90 T: *n* = 90	S: 36.7 (12.0) T: 36.4 (10.0)	S: 7.50 (1.30) T: 7.44 (1.20)	Size: 5~10 mm Location: distal	KUB, US, CT/ greatest dimension	Filter, US, CT	Diclofenac 50 mg prn Hydration: plenty of fluids	S: 8 mg qd T: 0.4 mg qd	3/3	4 weeks
Dell’Atti [23] 2015	RCT/Italy, 1 center	S: *n* = 68 T: *n* = 68	S: 36 (19~72) T: 35 (21~64)	S: 5.82 (1.66) T: 5.37 (1.33)	Size: 4~10 mm Location: distal	US, CT/ greatest dimension	Filter, KUB, US, CT	Diclofenac 100 mg, or paracetamol 1000 mg, or tramadol 100 mg prn Hydration: 2 L/day at least	S: 8 mg qd T: 0.4 mg qd	1/2	3 weeks
Georgescu [26] 2015	RCT/Romania, 1 center	S: *n* = 50 T: *n* = 50	S: 44.3 (13.0) T: 43.5 (13.3)	S: 5.32 (2.09) T: 5.08 (2.09)	Size: <10 mm Location: any	KUB, CT/ greatest dimension	Patient’s statement, KUB, US	Diclofenac 50 mg q12 h for 1 week and then q12 h prn Hydration: NA	S: 8 mg qd T: 0.4 mg qd	0/0	4 weeks
Elgalaly [24] 2016	RCT/Egypt, 1 center	S: *n* = 58 T: *n* = 57	S: 33.6 (9.9) T: 35.5 (11.3)	S: 5.4 (1.5) T: 5.6 (1.2)	Size: <10 mm Location: distal	KUB, US, CT/NA	Filter, KUB, US, CT	Diclofenac 50 prn Hydration: increased water intake	S: 8 mg qd T: 0.4 mg qd	6/6	4 weeks
AS [22] 2016	RCT/India, 1 center	S: *n* = 40 T: *n* = 40	S: 32 (7.5) T: 35 (8.5)	S: 7 (1.5) T: 7 (2)	Size: <10 mm Location: distal	KUB	Patient’s statement KUB, US, CT	Diclofenac 50~100 mg prn Hydration: NA	S: 8 mg qd T: 0.4 mg qd	0/0	4 weeks
Reddy [31] 2016	RCT/India, 1 center	S: *n* = 50 T: *n* = 50	S: 38 (21~70) T: 39 (21~70)	S: NA T: NA	Size: <10 mm Location: distal	KUB, US, CT/NA	Filter, KUB, US	Diclofenac 75 mg prn Hydration: 2 L/day at least	S: 8 mg qd T: 0.4 mg qd	0/0	4 weeks
Sharma [32] 2016	RCT/India, 1 center	S: *n* = 60 T: *n* = 60	S: NA T: NA	S: NA T: NA	Size: <10 mm Location: distal	KUB, US, CT/NA	Patient’s statement	Diclofenac dose: NA Hydration: NA	S: 8 mg qd T: 0.4 mg qd	8/6	4 weeks
Arda [33] 2017	Retrospective/ Turkey, 1 center	S: *n* = 159 T: *n* = 156	S: 45.9 (12.9) T: 44.6 (12.0)	S: 5.94 (1.23) T: 5.93 (1.07)	Size: 3~10 mm Location: distal	Radiographic images	KUB, US	NA Hydration: NA	S: 8 mg qd T: 0.4 mg qd	0/0	3 weeks
Fahmy [25] 2017	RCT/Egypt, 1 center	S: *n* = 30 T: *n* = 30	S: NA T: NA	S: NA T: NA	Children Size: <10 mm Location: distal	NA	NA	NA	S: 8 mg qd T: 0.4 mg qd	NA	4 weeks
Antony [21] 2017	RCT/India, 1 center	S: *n* = 79 T: *n* = 78	S: NA T: NA	S: NA T: NA	Size: <10 mm Location: any	KUB, CT/ greatest dimension	NA	NA	S: 8 mg qd T: 0.4 mg qd	NA	2 weeks
Rahman [29] 2017	RCT/India, 1 center	S: *n* = 40 T: *n* = 40	S: 34 (12) T: 38 (10)	S: 7.4 (1.3) T: 7.5 (1.2)	Size: 5~10 mm Location: distal	KUB / greatest dimension	KUB	Diclofenac 50 mg prn Hydration: plenty of fluids	S: 8 mg qd T: 0.4 mg qd	0/0	4 weeks
Priyanka [28] 2017	RCT/India, 1 center	S: *n* = 35 T: *n* = 35	S: 36.4 (12.7) T: 34.8 (12.7)	S: NA T: NA	Size: <10 mm Location: distal	US	US, KUB	NA	S: 8 mg qd T: 0.4 mg qd	8/7	4 weeks
Sentürk [35] 2018	Retrospective/Turkey, 1 center	S: *n* = 48 T: *n* = 48	S: 41.5 (15.0) T: 40.4 (12.4)	S: 6.65 (1.6) T: 7.10 (1.8)	Size: 4~10 mm Location: distal	CT	US/CT	NA	S: 8 mg qd T: 0.4 mg qd	0/0	4 weeks

RCT, randomized controlled trial; S, silodosin group; S, silodosin 4 mg/day group; T, tamsulosin group; NA, not available; KUB, kidney, ureter, and bladder plain radiograph; US, ultrasound; CT, computed tomography; SD, standard deviation; prn, pro re nata; qd, quaque dia.

### Risk of bias in the included studies

The quality and risk of bias of the included studies are listed in Table 2. For the RCT design, most studies had a low risk of randomization, incomplete outcome data, and reporting bias. Five [18, 21, 23, 24, 26] studies had a low risk of allocation and concealment. Five [18, 21, 23, 24, 26] of 13 RCTs had a low risk of performance bias. One study [28] had a high risk of attrition bias (incomplete outcome data). Seven studies [21, 22, 27–29, 31, 32] were rated as having a high risk of bias due to no prespecified sample size calculation. Three observational studies [33–35] were identified, and the quality of these studies was high (NOS score: 9).

**Table 2:** Risk of bias assessment for the included studies.

Cochrane risk of bias assessment for randomized controlled trials							
Study	Randomization	Allocation and concealment	Blinding of participant and study personnel	Blinding of outcome assessor	Incomplete outcome data	Reporting bias	Other bias
Gupta[27] 2013	Low*	Unclear	Low	Low	Low	Low	High^$^
Rathi[30] 2014	Unclear	Unclear	Unclear	Unclear	low	low	Unclear
Kumar[18] 2015	Low*	Low^#^	Low	Low	Low	Low	Low
Dell’Atti[23] 2015	Low*	Low^#^	Low	Low	Low	Low	Low
Georgescu[26] 2015	Low*	Low^#^	Low	Low	Low	Low	Low
Elgalaly[24] 2016	Low*	Low^#^	Low	Low	Low	Low	Low
AS[22] 2016	Unclear	Unclear	Unclear	Low	Low	Low	High^$^
Reddy[31] 2016	Low*	Unclear	Unclear	Low	Low	Low	High^$^
Sharma[32] 2016	Unclear	Unclear	Unclear	Unclear	Low	Low	High^$^
Fahmy[25] 2017	Unclear	Unclear	Unclear	Unclear	Low	Low	Unclear
Antony[21] 2017	Low*	Low^#^	Low	Unclear	Low	Low	High^$^
Rahman[29] 2017	Low*	Unclear	Unclear	Unclear	Low	Low	High^$^
Priyanka[28] 2017	Low*	Unclear	Unclear	Unclear	High	Low	High^$^

Newcastle-Ottawa scale^&^ (NOS) for assessment of observational studies										
Study, year	Selection				Comparability		Outcome			Total score
	Representativeness of the exposed cohort	Selection of the non-exposed cohort	Ascertainment of exposure	Demonstration that outcome of interest was not present at start of study	Comparability of cohorts on the basis of the design or analysis		Assessment of outcomes	Was follow-up long enough for outcomes to occur?	Adequacy of follow-up of cohorts	
					Age	Stone size				
Imperatore[34] 2014	Truly ★	Same community ★	Good ★	Yes ★	Yes ★	Yes ★	Yes ★	Yes ★	Yes ★	9
Arda[33] 2017	Truly ★	Same community ★	Good ★	Yes ★	Yes ★	Yes ★	Yes ★	Yes ★	Yes ★	9
Sentürk[35] 2018	Truly ★	Same community ★	Good ★	Yes ★	Yes ★	Yes ★	Yes ★	Yes ★	Yes ★	9

* Random number table;

^#^ sealed envelope;

^$^ no prespecified sample size calculation;

^★^ one star indicates 1 score;

^&^ NOS is a nine-point scale with a maximum of four points allocated to selection, two points for comparability, and three points for outcome. Studies scoring ≥7 are considered high quality, 4~6, moderate quality, and ≤4, low quality.

### Primary outcomes

#### 1. Expulsion rate (at the end of the study)

**Fig 2:**
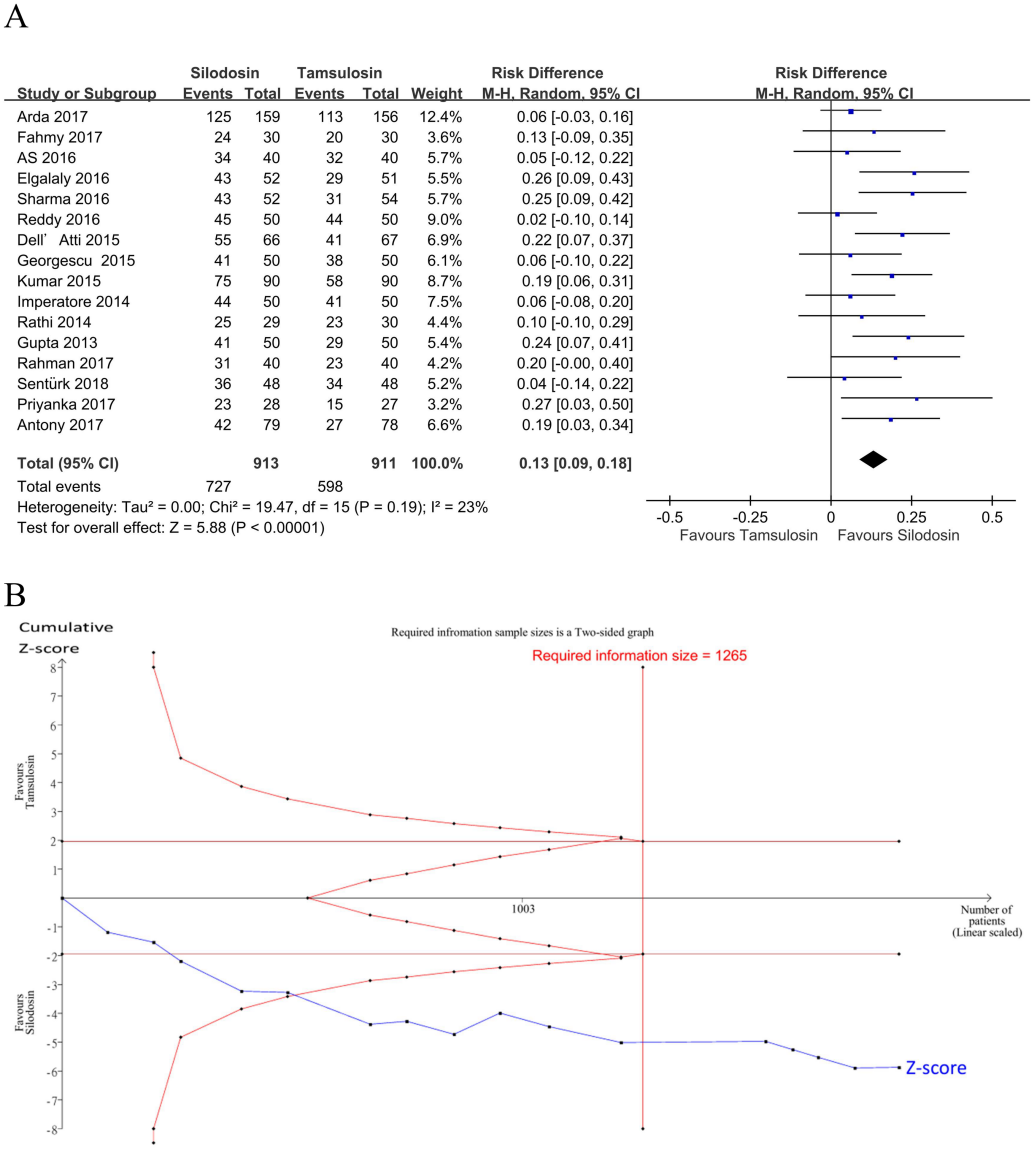
Forest plot and trial sequential analysis for the stone expulsion rate. A: Forest plot. RD, risk difference; CI, confidence interval. B: Trial sequential analysis for the effect of silodosin on the expulsion rate compared to tamsulosin. The risk of a type I error was maintained at 5% with 90% power. The variance was calculated from data obtained from the trials included in this meta-analysis. A clinically meaningful intervention effect for stone expulsion was set to a 15% relative risk reduction based on the assumption of a 65% proportion of the control group. The result showed that solid evidence indicated that silodosin had a higher expulsion rate compared to tamsulosin.

Sixteen studies [18, 21–35] (*n* = 1824, thirteen RCTs and three observational studies) evaluated the expulsion rate at the end of the study (Fig 2A). Silodosin achieved significantly higher expulsion rates than tamsulosin (pooled RD: 0.13, 95% CI: 0.09 to 0.18, I^2^ = 23%), such that eight patients would need treatment for one patient to realize a benefit from silodosin.

A subgroup analysis showed that the expulsion rate changed with stone size and study design and was not influenced by the follow-up time (1, 2, 3, or 4 weeks) or stone location (only DUSs or not only DUSs) (Table 3). For stone sizes, silodosin had a significantly higher expulsion rate than tamsulosin in patients with stone sizes of 5~10 mm (five studies [18, 26, 29, 31, 34], *n* = 410, pooled RD: 0.14, 95% CI: 0.06 to 0.22, I^2^ = 0%). However, silodosin had no superior effect over tamsulosin for stone sizes of <5 mm (three studies [26, 31, 34], *n* = 150, pooled RD: 0.03, 95% CI: -0.06 to 0.11, I^2^ = 0%). For the study design, silodosin had a significantly higher expulsion rate than tamsulosin in RCTs (13 studies [18, 21–32], *n* = 1313, pooled RD: 0.16, 95% CI: 0.11 to 0.21, I^2^ = 16%). High stone expulsion rates were observed in observational studies, but the effect size was smaller than with RCTs and was not significant (three studies [33–35], *n* = 514, pooled RD: 0.06, 95% CI: -0.01 to 0.13, I^2^ = 0%).

**Table 3:** Predefined clinical subgroup analysis with expulsion rate comparing silodosin with tamsulosin.

Category	Subgroups	No of studies	No of patients	RD [95% CI]	p value	Group heterogeneity		Subgroup difference	
						I^2^	P value	I^2^	p value
Outcome: Expulsion rate									
All study		14	1673	0.13 [0.09, 0.18]	<0.05	25	0.19	NA	
Stone size	<5 mm	3	150	0.03 [-0.06, 0.11]	0.52	0	0.84	69	<0.05*
	5–10 mm	5	410	0.14 [0.06, 0.22]	<0.05	0	0.64		
Stone location	Only distal	13	1467	0.13 [0.08, 0.18]	<0.05	26	0.18	0	0.67
	Not only distal	3	357	0.16 [0.06, 0.26]	<0.05	19	0.29		
Follow up	1 week	3	551	0.11 [0.04, 0.19]	<0.05	69	<0.05	0	0.55
	2 weeks	4	708	0.19 [0.12, 0.26]	<0.05	89	<0.05		
	3 weeks	3	551	0.14 [0.06, 0.21]	<0.05	64	0.06		
	4 weeks	13	1165	0.15 [0.10, 0.20]	<0.05	22	0.21		
Study design	RCT	13	1313	0.16 [0.11–0.21]	<0.05	16	0.28	80	<0.05*
	Observational study	3	514	0.06 [-0.01, 0.13]	0.11	0	0.98		

CI, confidence interval; RCT, randomized control trial;

*, statistically significant.

**Fig 3:**
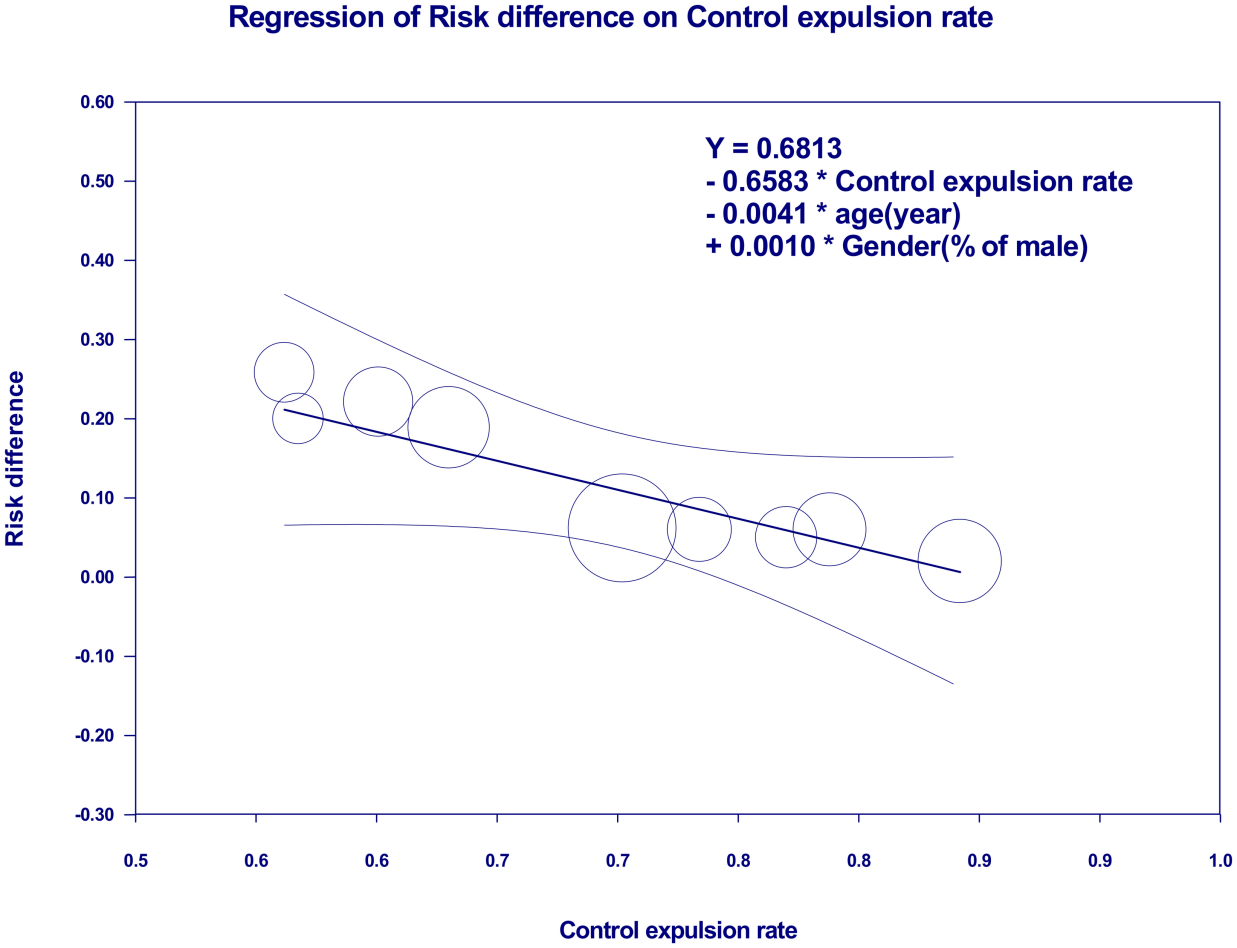
Meta-regression for the risk difference (RD) of stone expulsion rates between silodosin and tamsulosin. The RD was proportional to the control expulsion rate.

To determine whether the effect size varied with age, gender, stone sizes, laterality of stone location, or the control expulsion rate, we performed a meta-regression. The RD for silodosin compared to tamsulosin was not moderated by gender (*p* = 0.35), stone size (*p* = 0.89), or laterality of the stone location (*p* = 0.13) according to a univariate regression model (Table 4). However, age (*p* = 0.04) and the control expulsion rate (*p* = 0.001) had negative associations with the RD for stone expulsion (Table 4). After adjusting for either age, gender, or both, the negative association between the RD and the control expulsion rate remained (Table 5, model 1–3). However, the negative association between the RD and age was not observed after adjusting for the control expulsion rate (Table 5, models 2–3). In model 3, the best model for predicting the association with effect sizes after adjusting for age and gender (adjusted *R*^2^ = 1.0), for every 10% increase in the baseline risk, the risk difference of stone passage decreased by 6.58% (Fig 3).

**Table 4:** Univariate meta-regression predicting estimates of the expulsion rate.

Covariate	No of study	Univariate analysis		
		Coefficients (95% CI)	p-value	Adjusted R2
Gender (% of male)	12	-0.002 (-0.005~0.002)	0.35	-10
Age (years)	11	-0.009 (-0.017~-0.0002)	0.04*	81
Stone size	10	0.006 (-0.070~0.081)	0.89	-47
Stone location (Laterality)	6	0.007 (-0.002~0.015)	0.13	42
Control expulsion rate	16	-0.499 (-0.795~-0.202)	0.001*	100

*, statistically significant.

**Table 5:** Multivariate meta-regression models predicting estimates of the expulsion rate.

Covariate	Multivariate analysis								
	Model 1 (No of study = 10)			Model 2 (No of study = 9)			Model 3 (No of study = 9)		
	Coefficient (95% CI)	p value	Adjusted R2	Coefficient (95% CI)	p value	Adjusted R2	Coefficient (95% CI)	p value	Adjusted R2
Gender (% of male)	0.0001(-0.003~0.003)	0.95	100.00	NA	NA	NA	0.001 (-0.003~0.005)	0.63	100.00
Age (years)	NA	NA	NA	-0.003 (-0.017~0.006)	0.55	100.00	-0.004(-0.015~0.007)	0.45	
Control expulsion rate	-0.762(-1.210~-0.314)	<0.05*	100.00	-0.680(-1.189~-0.171)	<0.05*		-0.658 (-1.175~-0.142)	<0.05*	

NA, no analysis;

*, statistically significant.

A sensitivity analysis was used to test the robustness of our findings based on RCTs excluded because of the abstract, those excluded because of a high or unclear risk in each domain of the risk of bias, those excluded because of unclear information about age or stone sizes, and those excluded because of unclear information for the measurement of stone passage and hydration. These factors did not influence our findings (Table 6). The outcome measure cross-validated using the RR or the OR also showed the robustness of our findings.

**Table 6:** Sensitivity analyses: The effect of potential biases on primary outcomes.

Potential bias or limitations excluded	No of studies	No of patients	RD (95% CI)	I2 (%)	p value	RR (95% CI)	I2 (%)	p value	OR (95% CI)	I2 (%)	p value
Overall	16	1824	0.13 (0.09–0.18)	23	<0.05	1.19(1.11–1.28)	39	<0.05	2.11(1.70–2.63)	0	<0.05
RCT	13	1313	0.16 (0.11–0.21)	16	<0.05	1.24(1.14–1.36)	43	<0.05	2.49(1.92–3.22)	0	<0.05
RCT exclude abstract	11	1194	0.17 (0.11–0.22)	28	<0.05	1.26(1.14–1.40)	52	<0.05	2.54(1.94–3.33)	0	<0.05
RCT quality^a^											
Randomization	9	1008	0.17 (0.11–0.23)	27	<0.05	1.28(1.14–1.44)	54	<0.05	2.54(1.90–3.40)	0	<0.05
Allocation and concealment	5	673	0.18 (0.12–0.25)	0	<0.05	1.29(1.15–1.46)	22	<0.05	2.52(1.78–3.56)	0	<0.05
Blinding of participant and study personnel	6	773	0.19 (0.13–0.25)	0	<0.05	1.31(1.18–1.44)	9	<0.05	2.60(1.88–3.61)	0	<0.05
Blinding of outcome assessor	7	796	0.14 (0.07–0.22)	46	<0.05	1.21(1.07–1.36)	58	<0.05	2.47 (1.75–3.48)	0	<0.05
Incomplete outcome data	12	1258	0.15 (0.10–0.21)	18	<0.05	1.23(1.13–1.35)	45	<0.05	2.44(1.87–3.18)	0	<0.05
Reporting bias	11	1152	0.15 (0.10–0.21)	16	<0.05	1.21(1.11–1.34)	44	<0.05	2.36(1.79–3.10)	0	<0.05
Other bias	4	516	0.18 (0.10–0.26)	10	<0.05	1.27(1.13–1.44)	24	<0.05	2.69(1.78–4.07)	0	<0.05
Participants^b^											
Age	11	1342	0.12 (0.06–0.17)	30	<0.05	1.16(1.07–1.25)	38	<0.05	1.96(1.51–2.55)	0	<0.05
Stone size	10	1287	0.13 (0.08–0.19)	26	<0.05	1.18(1.09–1.28)	32	<0.05	2.03(1.56–2.64)	0	<0.05
Method^c^											
Measurement of stone passage	13	1548	0.13 (0.08–0.19)	36	<0.05	1.19(1.10–1.29)	45	<0.05	2.12(1.67–2.70)	0	<0.05
Hydration	8	1117	0.14 (0.08–0.21)	48	<0.05	1.21(1.09–1.34)	57	<0.05	2.24(1.66–3.03)	8	<0.05

^a,^ excluded high or unclear risk;

^b,^ excluded with unclear information for age or stone size;

^c,^ excluded with unclear information for measurement of stone passage and hydration;

CI, confidence interval; N/A, not applicable; RD, risk difference; RR, risk ratio; OR, odds ratio.

**Fig 4:**
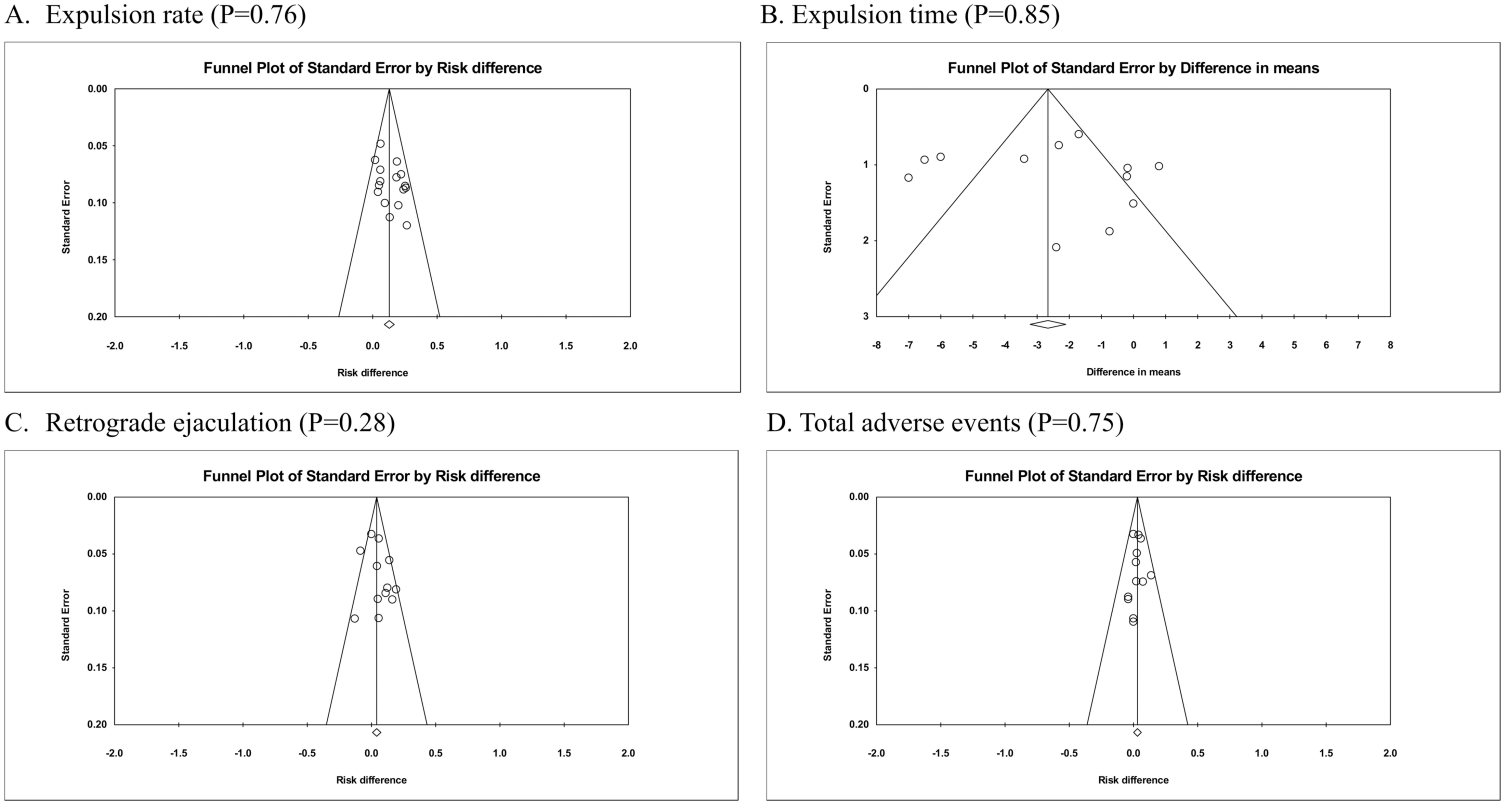
Funnel plots of comparisons of silodosin with tamsulosin. A: expulsion rate, B: expulsion time, C: retrograde ejaculation, and D: total adverse events.

Inspection of the funnel plots showed no asymmetry (Fig 4A, Egger’s test: *p* = 0.76), indicating no evidence of a small study effect.

In the TSA, the Z-curve crossed the TSA monitoring boundary (Fig 2B). The TSA-adjusted CI was 0.09 to 0.18. The accrued information size (*n* = 1824) reached the RIS (*n* = 1265). The GRADE was rated high because no serious risk of bias, inconsistence, indirectness, imprecision, or publication bias was detected (Table 7).

**Table 7:** The GRADE evidence quality for main outcomes.

Certainty assessment							Summary of findings				
№ of participants (studies)	Risk of bias	Inconsistency	Indirectness	Imprecision	Publication bias	Overall certainty of evidence	Study event rates (%)		Relative effect (95% CI)	Anticipated absolute effects	
							With tamsulosin	With silodosin		Risk with tamsulosin	Risk difference with silodosin
**Expulsion rate**											
1313 (13 RCTs)	not serious^a^	not serious	not serious	not serious^e^	none	⨁⨁⨁⨁ HIGH	410/657 (62.4%)	522/656 (79.6%)	**RR 1.24** (1.14 to 1.36)	**Study population**	
										624 per 1000	**150 more per 1000** (87 more to 225 more)
										**Low**	
										350 per 1000	**84 more per 1000** (49 more to 126 more)
										**High**	
										880 per 1000	**211 more per 1000** (123 more to 317 more)
**Expulsion time**											
983 (10 RCTs)	not serious^b^	serious^d^	not serious	not serious^e^	none	⨁⨁⨁◯ MODERATE	502	481	-	The mean expulsion time ranged 6.4~21 days	MD 2.80 days lower (4.62 lower to 0.99 lower)
**Pain episodes**											
708 (7 RCTs)	serious^c^	serious^d^	not serious	not serious	none	⨁⨁◯◯ LOW	355	353	-	The mean pain episodes ranged 1.4~3.1 episodes	MD 0.33 episodes lower (0.57 lower to 0.10 lower)
**Requirement of analgesic**											
383 (3 RCTs)	serious^c^	serious^d^	not serious	serious^f^	none	⨁◯◯◯ VERY LOW	191	192	-	-	SMD 0.90 lower (2.36 lower to 0.56 higher)
**Retrograde ejaculation**											
697 (10 RCTs)	serious^c^	not serious	not serious	serious^f^	none	⨁⨁◯◯ LOW	28/344 (8.1%)	53/353 (15.0%)	RR 1.61 (0.98 to 2.65)	Study population	
										81 per 1000	50 more per 1000 (2 fewer to 134 more)
										Low	
										0 per 1000	0 fewer per 1000 (0 fewer to 0 fewer)
										High	
										280 per 1000	171 more per 1000 (6 fewer to 462 more)
**Postural hypotension**											
835 (9 RCTs)	serious^c^	not serious	not serious	serious^f^	none	⨁⨁◯◯ LOW	23/418 (5.5%)	16/417 (3.8%)	RR 0.71 (0.37 to 1.34)	Study population	
										55 per 1000	16 fewer per 1000 (35 fewer to 19 more)
										Low	
										0 per 1000	0 fewer per 1000 (0 fewer to 0 fewer)
										High	
										83 per 1000	24 fewer per 1000 (52 fewer to 28 more)
**Total Adverse effect**											
1041 (10 RCTs)	serious^c^	not serious	not serious	serious^f^	none	⨁⨁◯◯ LOW	104/522 (19.9%)	123/519 (23.7%)	RR 1.12 (0.91 to 1.31)	Study population	
										199 per 1000	24 more per 1000 (18 fewer to 78 more)
										Low	
										0 per 1000	0 fewer per 1000 (0 fewer to 0 fewer)
										High	
										430 per 1000	52 more per 1000 (39 fewer to 168 more)

CI, confidence interval; RR, risk ratio; MD, mean difference; SMD, standardized mean difference; RCT, randomized control trial;

^⨁⨁⨁⨁,^ high-grade recommendation;

^⨁⨁⨁◯,^ moderate-grade recommendation;

^⨁⨁◯◯,^ low-grade recommendation;

^⨁◯◯◯,^ very low-grade recommendation.

^a.^ The result of the sensitivity analysis showed that each domain of risk of bias did not influence our finding.

^b.^ The result of the subgroup analysis showed that expulsion time was not influenced by the study design.

^c.^ Most trials were rated as having an unclear or high risk of bias.

^d.^ High I^2^ values.

^e.^ Trial sequential analysis indicated that the required information sizes were reached.

^f.^ Wide confidence interval, trial sequential analysis indicated that the required information sizes were not reached.

### Secondary outcomes

#### 1. Expulsion time (days)

**Fig 5:**
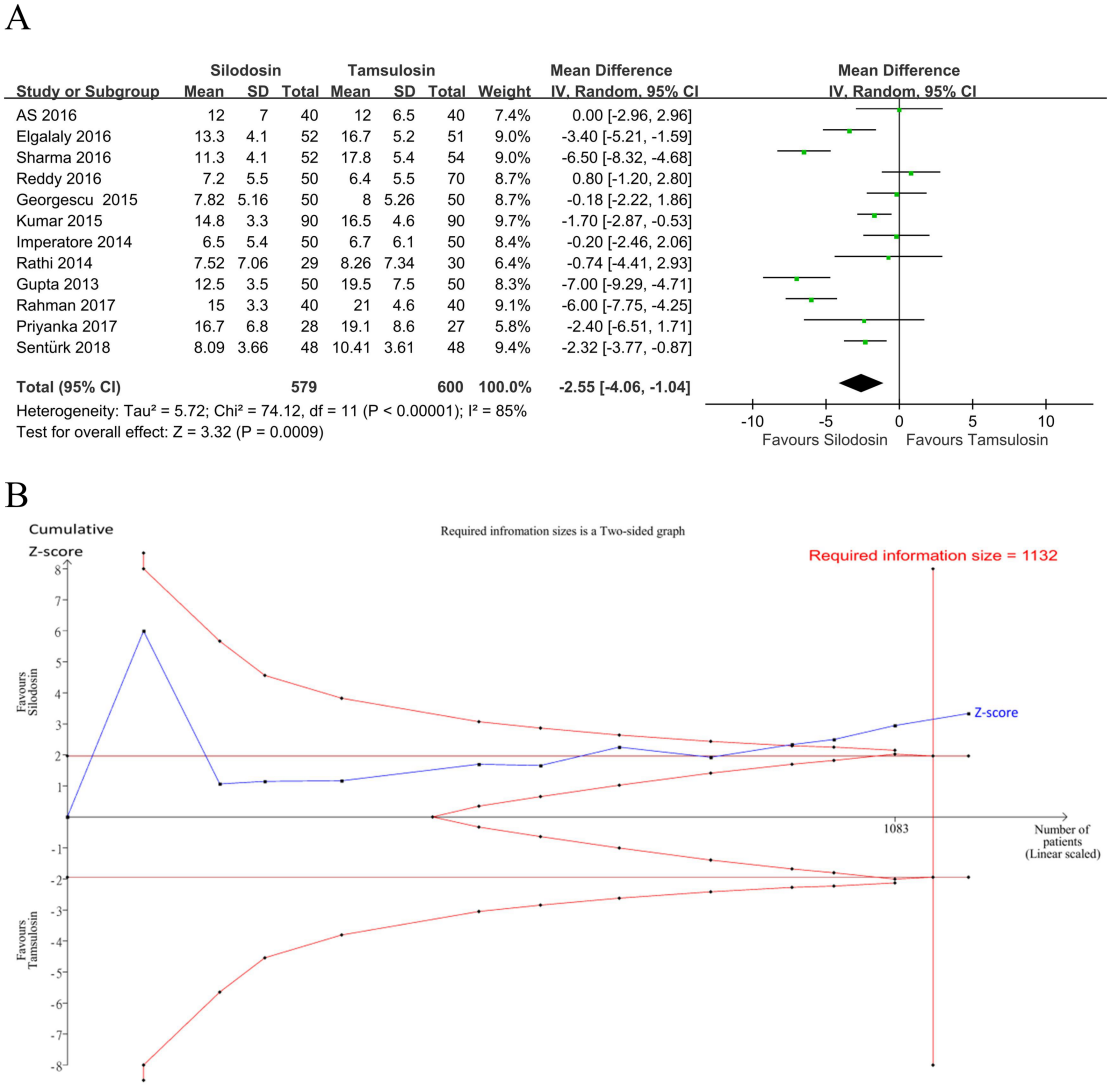
Forest plot and trial sequential analysis for stone expulsion times. A: Forest plot. MD, mean difference; CI, confidence interval. B: Trial sequential analysis of the effect of silodosin on the expulsion time compared to tamsulosin. The risk of a type 1 error was maintained at 5% with a power of 90%. The variance was calculated from data obtained from the included trials. A clinically significant anticipated mean difference in expulsion times was set to 2.55 days based on the pooled result of our meta-analysis. The result showed that solid evidence indicated that silodosin had a shorter expulsion time compared to tamsulosin.

Twelve studies [18, 22, 24, 26–32, 34, 35] (*n* = 1179, ten RCTs and two observational studies) evaluated the expulsion time (Fig 5A). Compared to tamsulosin, patients who received silodosin had a significantly shorter time for stone expulsion (PMD: -2.55 days, 95% CI: -4.06 to -1.04, I^2^ = 85%). However, an I^2^ test of >60% indicated high heterogeneity, indicating that caution needs to be taken when interpreting these results. A subgroup analysis showed that the expulsion time was not influenced by the stone size (>5 or <5 mm), stone location (only DUSs or not only DUSs), or study design (Table 8). Inspection of the funnel plots showed no asymmetry (Fig 4B, Egger’s test: *p* = 0.85), indicating no evidence of a small study effect. In the TSA, the Z-curve crossed the TSA monitoring boundary (Fig 5B). The TSA-adjusted CI was -4.20 to -0.90 (D^2^ = 86%). The accrued information size (*n* = 1179) reached the RIS (*n* = 1132). The GRADE was rated moderate because no serious risk of bias, indirectness, imprecision or publication bias, except inconsistency, was detected (Table 7).

**Table 8:** Predefined clinical subgroup analysis with expulsion time comparing silodosin with tamsulosin.

Category	Subgroups	No of studies	No of patients	RD (95% CI)	P value	Group heterogeneity		Subgroup difference	
						I^2^	P value	I^2^	P value
Outcome: Expulsion time									
All study		12	1179	-2.55 [-4.06, -1.04]	< 0.05	85	< 0.05		
Stone size	<5 mm	2	100	0.49 [-0.15, 1.14]	0.13	67	0.08	0	0.32
	>5 mm	2	100	0.11 [-0.27, 0.50]	0.56	0	0.77		
Stone location	Only distal	10	979	-2.37 [-3.92, -0.81]	< 0.05	84	< 0.05	0	0.73
	Not only distal	2	200	-3.57 [-10.25, 3.11]	0.30	95	< 0.05		
Study design	RCT	10	983	-2.80 [-4.72, -0.99]	< 0.05	86	< 0.05	0	0.33
	Observational study	2	196	-1.44 [-4.06, 1.04]	0.17	58	0.12		

RD, risk difference; CI, confidence interval; RCT, randomized control trial.

#### 2. Pain episodes

**Fig 6:**
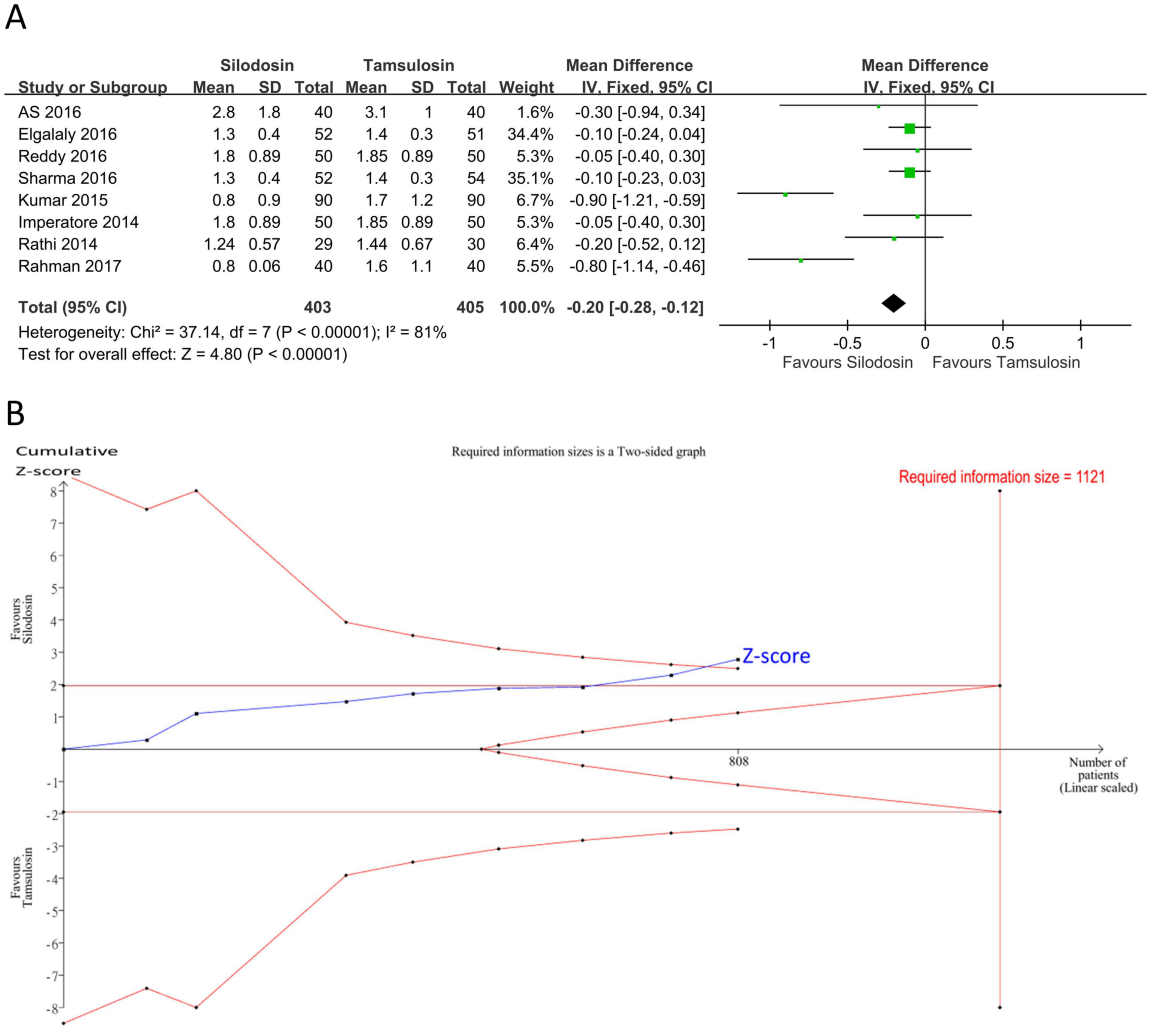
Forest plot and trial sequential analysis for pain episodes. A: Forest plot. MD, mean difference; CI, confidence interval. B: Trial sequential analysis of the effect of silodosin on pain episodes compared to tamsulosin. The risk of a type 1 error was maintained at 5% with a power of 90%. The variance was calculated from the data obtained from the included trials. A clinically significant anticipated mean difference in the expulsion time was set to 0.3 episodes based on the pooled result of our meta-analysis. The result showed that firm evidence indicated that silodosin had fewer pain episodes compared to tamsulosin.

Eight studies [18, 22, 24, 29–32, 34] (*n* = 808, seven RCTs and one observational study) evaluated pain episodes (Fig 6A). The results indicated that silodosin had fewer pain episodes than tamsulosin (PMD: -0.3 episodes, 95% CI: -0.51 to -0. 09, I^2^ = 81%). Heterogeneity was high. A funnel plot was not created because there were fewer than 10 studies included. The TSA-adjusted CI was -0.57 to -0. 03 (D^2^ = 86%). The Z-curve crossed the TSA monitoring boundary (Fig 6B). The accrued information size (*n* = 808) was 72% of the RIS (*n* = 1121). The GRADE was rated low because a serious risk of bias and inconsistency was detected (Table 7).

#### 3. Requirement for analgesics

**Fig 7:**
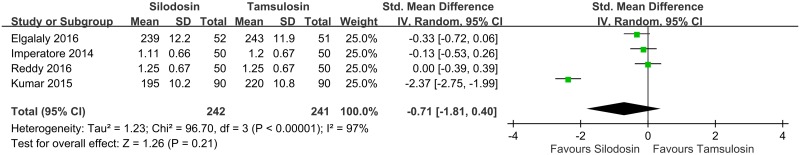
Forest plot of requirement for analgesics.

Four studies [18, 24, 31, 34] (*n* = 483, three RCTs and one observational study) evaluated the requirement for analgesics (Fig 7). The pooled SMD indicated no significant difference favoring silodosin compared to tamsulosin (pooled SMD: -0.71, 95% CI: -1.81 to 0.40, I^2^ = 97%). The results demonstrated that a small sample size caused imprecision in estimating the effect and that the heterogeneity was high. A funnel plot was not created because fewer than 10 studies were included. The GRADE was rated low because a serious risk of bias, inconsistency, and imprecision was detected (Table 7).

#### 4. Safety outcomes

**Fig 8:**
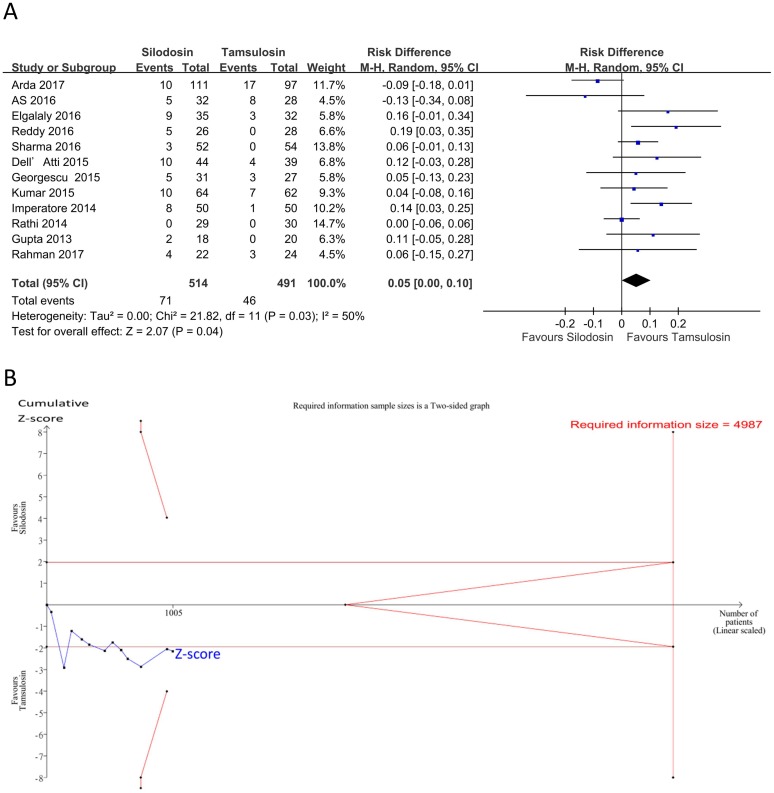
Forest plot and trial sequential analysis for retrograde ejaculation. A: Forest plot. RD, risk difference; CI, confidence interval. B: Trial sequential analysis assessing the effect of silodosin versus tamsulosin on retrograde ejaculation. The risk of a type I error was maintained at 5% with 90% power. The variance was calculated from data obtained from the trials included in this meta-analysis. A clinically meaningful intervention effect for stone expulsion was set to a 50% relative risk reduction based on the assumption of 9.4% proportion of the control group.

**Fig 9:**
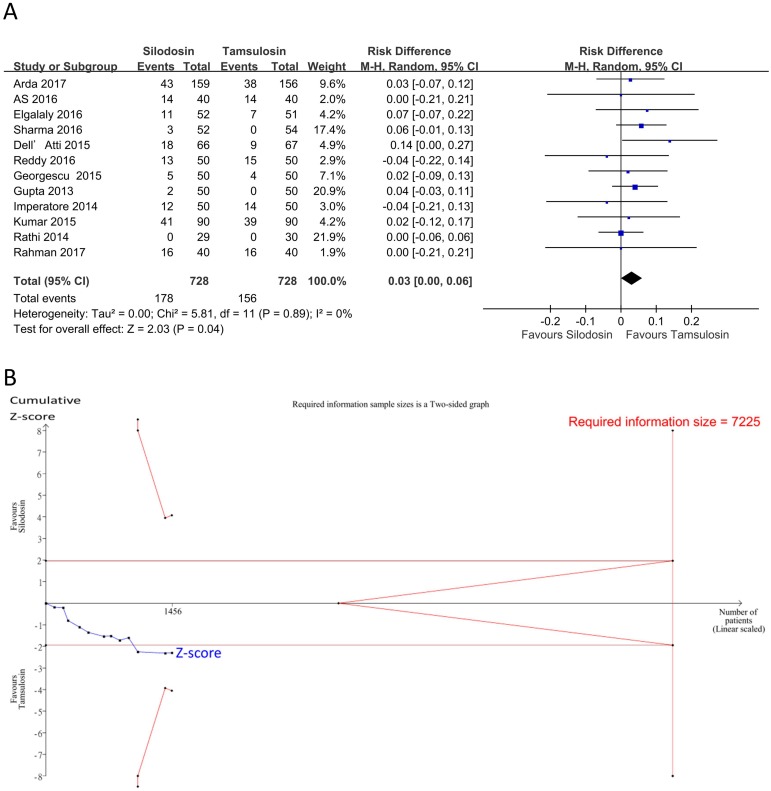
Forest plot and trial sequential analysis for total adverse events. A: Forest plot. RD, risk difference; CI, confidence interval. B: Trial sequential analysis assessing the effect of silodosin versus tamsulosin on total adverse events. The risk of a type I error was maintained at 5% with 90% power. The variance was calculated from data obtained from trials included in this meta-analysis. A clinically meaningful intervention effect was set to a 15% relative risk reduction based on the assumption of a 21.4% proportion of the control group.

Results of the meta-analysis on safety outcomes are summarized in Table 9. Our pooled results of limited studies showed no significant and minimal risk differences of adverse effects, including postural hypotension, headaches, dizziness, backache, gastrointestinal effects, or revisits, between silodosin and tamsulosin. However, silodosin had a significantly higher incidence of retrograde ejaculation than tamsulosin by 5% and a higher incidence of total adverse events by 3% (Figs 8A and 9A; retrograde ejaculation: 12 studies [18, 22–24, 26, 27, 29–34], *n* = 1005, RD = 0.05,95% CI: 0.00 to 0.10, I^2^ = 50%; total adverse events: 12 studies [18, 22–24, 26, 27, 29–34], *n* = 1456, RD = 0.03,95% CI: 0.00 to 0.06, I^2^ = 0%). Inspection of the funnel plots showed no asymmetry (Fig 4C and 4D, Egger’s test: retrograde ejaculation (*p* = 0.28); total adverse events (*p* = 0.75)), indicating no evidence of a small study effect. In the TSA for retrograde ejaculation and total adverse events, the Z-curve did not cross the TSA monitoring boundary or the futility boundary (Figs 8B and 9B). The TSA-adjusted CI was -0.05 to 0.16 on retrograde ejaculation and -0.03 to 0.09 on total adverse events, indicating the imprecision of the study. The accrued information size did not reach the RIS on retrograde ejaculation or total adverse events. The GRADE was rated low because of a serious risk of bias and imprecision (Table 7).

**Table 9:** Summary of results of the meta-analysis of safety outcomes.

Outcome	No. of studies	No. of points	Pooled effects, RD (95% CI)	Analytical model	*p*	I^2^ (%)
Silodosin vs. tamsulosin						
Retrograde ejaculation	12	1005	RD, 0.05 (0.00 to 0.10)	Random	0.04*	50
Postural hypotension	11	1476	RD, -0.01 (-0.03 to 0.02)	Random	0.65	33
Headache	7	967	RD, -0.01 (-0.03 to 0.02)	Random	0.49	0
Dizziness	6	652	RD, -0.02 (-0.05 to 0.02)	Random	0.32	0
Backache	3	319	RD, 0.01 (-0.04 to 0.05)	Random	0.74	0
Nasal congestion	3	259	RD, -0.00 (-0.04 to 0.04)	Random	0.85	0
GI effect	3	272	RD, 0.01 (-0.02 to 0.04)	Random	0.49	0
Revisit	2	180	RD, 0.02 (-0.04 to 0.08)	Random	0.49	0
Total adverse effect	12	1456	RD, 0.03 (0.00 to 0.6)	Random	0.04*	0

RD, risk difference; CI, confidence interval;

*, statistically significant.

## Discussion

In this comprehensive systematic review and meta-analysis, we included 16 studies with 1812 patients. The results showed that silodosin helped 13% more patients facilitate the passage of ureteric stones, particularly those that were >5 mm in size, compared with Tamsulosin, regardless of their age, gender, or stone location. The TSA further provided a sufficient level of evidence with the power of accuracy and reliability for the meta-analysis. Additionally, silodosin therapy for ureteral stones probably had a shorter expulsion time by 2.55 days and may have fewer colic episodes than tamsulosin. However, the use of silodosin may have a higher risk of retrograde ejaculation by 5% and total adverse events by 3% compared with tamsulosin.

The aim of MET is to facilitate the spontaneous passage of ureteral stone. However, the use of MET has been debated due to controversial results between meta-analyses pooling results from RCTs and recent multicenter RCTs. In 2015, Pickard et al. [36] conducted a multicenter RCT in the United Kingdom that included over 1100 patients comparing tamsulosin, nifedipine, and placebo for ureteral stones <10 mm. The results showed that no difference was observed at the need for intervention for stone clearance. However, this study was underpowered for stones >5 mm (25% of all stones were >5 mm), and concerns have been raised about the high baseline rate of spontaneous stone passage. In 2016, Furyk et al. [37] conducted a multicenter RCT in Australia. The result identified a benefit for using MET for stones sized >5 mm but no effect for stones <5 mm. In 2017, Ye et al. [38] conducted a multicenter RCT in China (all stones, n = 3296; stones >5 mm, n = 1116). The results showed tamsulosin benefits the expulsion of distal ureteral stones >5 mm. By contrast, Hollingsworth et al. [39] conducted a systematic review, identifying all randomized controlled trials examining alpha blockers for the treatment of ureteric stones. They concluded that MET is effective in patients with ureteric stones who are amenable to conservative management, which was supported by another review conducted by Skolarikos et al. [40] in 2017. Taken together, there is sufficient evidence to support the clinical use of MET for the management of ureteral stones >5 mm.

Several previous systematic reviews and meta-analyses investigated silodosin versus tamsulosin for the treatment of ureteral stones [7–11]. The most recent study by Liu and colleagues [9] identified only five RCTs that yielded a pooled risk ratio of 1.25 (95% CI: 1.13 to 1.37), favoring silodosin over tamsulosin. Our meta-analysis also showed similar results (RR: 1.19, 95% CI: 1.11 to 1.28). However, an additional analysis, the sensitivity analysis, and the detection of publication bias was not addressed by Liu and colleagues [9] due to the limited number of identified studies. However, we were able to address these issues. We did not detect publication bias for the included studies. In the TSA of expulsion rates, the accrued information size reached the RIS, indicating that our finding was powerful. Furthermore, our findings showed that international differences in control expulsion rates influenced the risk differences of stone passage, which was supported by a report by Hollingsworth et al. [39]. The explanation was that patient-related factors could modify the effects of expulsive therapy. To clarify the issue, future researchers should consider including variables such as patient age, gender, race/ethnicity, computed tomographic findings, and detailed information about subgroups with different stone sizes and laterality of stones in the design of large international trials.

Regarding stone size, our results showed that there were no differences in stone expulsion rates between silodosin and tamsulosin in patient with smaller stones (<5 mm). Given that 95% of stones of <4 mm passed within 40 days [41], MET in this subgroup likely provided only a minimal effect, and this observation is reasonable for small stones, which is consistent with guidelines of the European Association of Urology [5]. For larger stones (5~10 mm), most network meta-analyses focused on different medications for MET, and those findings suggested that α-blockers had the highest ranking for MET [6, 39, 42]. However, which type of α-blocker provides the greatest benefits to patients with large stones was not clarified. Even the most recent meta-analysis by Liu and colleagues [9] did not clarify this issue due to the limited number of included studies. Our results showed that silodosin provided a high stone expulsion rate of 14% over tamsulosin for larger stones. The explanation is that α-adrenergic receptors are classified into three different subtypes of α-1A, α-1B, and α-1D, and the distribution in the human ureter is α-1D > α-1A > α-1B receptors [43]. Based on their findings, an α-1D-adrenoceptor blocker may provide better stone expulsion than an α-1A-adrenoceptor blocker. However, ureteral contractions were mainly mediated by α-1A-adrenoceptors in a hamster study [44]. Tsuzaka and colleagues [45] reported that an α-1A-adrenoceptor blocker provided more stone expulsions than an α-1D-adrenoceptor blocker. Silodosin had an equal affinity for the α-1D subtype as tamsulosin, but the affinity of silodosin for the α-1A subtype was approximately 17-fold greater than tamsulosin [8]. This is the reason that silodosin provided better stone clearance than tamsulosin.

Regarding expulsion times, our results showed that mean expulsion times with tamsulosin ranged from 6.4~21 days, whereas mean expulsion times with silodosin ranged from 6.5~16.7 days. Silodosin probably provided shorter expulsion times by approximately 3 days compared with tamsulosin, but high heterogeneity was found. Elgalaly et al. [24] reported that several factors can affect the time to expulsion, such as the stone size, site, presence or absence of ureteric smooth muscle spasms, and submucosal edema. However, stone size and stone location cannot explain the heterogeneity of our subgroup analyses. Thus, there may have been methodological and clinical reasons for the heterogeneity among the included studies.

Another important clinical consideration is that, theoretically, although the increase in the stone clearance rate and faster stone expulsion times would allow less analgesic requirements, our analysis found that silodosin may have comparable efficacy for the requirement of analgesics as tamsulosin. The reason is that limited original publications reported this outcome, and thus we could not precisely determine whether silodosin has fewer requirements for analgesics than tamsulosin.

For adverse events, we found no risk differences of common adverse effects, including headaches, dizziness, backache, nasal congestion, gastrointestinal effects, and postural hypotension, between silodosin and tamsulosin. By contrast, retrograde ejaculation was another common major side effect, and previous meta-analyses also showed that there was no significant difference between silodosin and tamsulosin in terms of retrograde ejaculation [7–11]. However, in the present meta-analysis, the results showed that silodosin may have a higher risk by 5% compared with tamsulosin for retrograde ejaculation, which contributed to higher total adverse events in the silodosin group. One possible explanation is that we included more studies with more patients and provided a more-precise estimate than previous studies. Jung et al. [46] conducted a Cochrane review to assess the effects of silodosin for the treatment of lower urinary tract symptoms in men with benign prostatic hyperplasia. The result showed that silodosin likely increased sexual adverse events compared to tamsulosin, naftopidil or alfuzosin (follow up ≤ 12 weeks). However, most studies included in the current study reported that retrograde ejaculation did not interrupt the intervention and was reversible after withdrawal from treatment [18, 22–24, 26, 27, 29–34]. Thus, the use of silodosin for MET is considered safe.

The present meta-analysis has some limitations. Given that the inclusion of observational studies could lead to a wrong estimation of the true intervention effect, we conducted subgroup analyses, which showed that the RD of the stone expulsion rate was significantly higher in RCT designs. A higher stone expulsion rate was observed with silodosin than with tamsulosin in the observational studies, although the effect was not significant. This may be related to the small sample sizes of the observational studies to demonstrate an effect. Because the overall methodological rigor of the pooled studies may have limited application of our findings, we performed a sensitivity analysis according to each domain of the risk of bias for RCTs. The results showed that our findings were robust. Since the different degrees for detecting stone passage in the included studies may have biased the estimate, we excluded unclear information or the detection of stone passage only by patient reports. The findings of our sensitivity analyses did not change. Because concomitant pain management regimes differed among the studies, we did not clarify the influence on the pooled results. In addition, the currently available evidence has insufficient power to address the effect on colic episodes, retrograde ejaculation, and total adverse events. Finally, although our results were not affected by publication bias, we predict that some smaller studies with negative results were not published.

In conclusion, compared to tamsulosin, silodosin provided significantly higher stone expulsion rates, particularly for stone sizes of 5~10 mm. Silodosin may also have benefits of shorter stone expulsion times and fewer colic episodes than tamsulosin. However, this may be at the expense of increased adverse events such as retrograde ejaculation.

## Supporting information

S1 Checklist PRISMA checklist. (DOC)

S1 Table Search strategy. (DOCX)

S2 Table Baseline characteristics of the included studies. (DOCX)
