# Supplementary material for: Silodosin versus tamsulosin for medical expulsive treatment of ureteral stones: A systematic review and meta-analysis
Source: PLoS One. 2018 Aug 28;13(8):e0203035. doi: 10.1371/journal.pone.0203035 (PMC6112672; doi:10.1371/journal.pone.0203035)
Supplement: S1 Table — (DOCX) [file pone.0203035.s002.docx]

**S1Table Search Strategy**

| Recent queries in pubmed on May 12, 2018 | | | |
| --- | --- | --- | --- |
| Search | Query | Items found | |
| 1 | urolithiasis | 37901 | |
| 2 | ureterolithiasis | 6421 | |
| 3 | ureteral calculi | 8538 | |
| 4 | nephrolithiasis | 20760 | |
| 5 | colic | 10452 | |
| 6 | stone* or calculi* or colic and ureter* | 109310 | |
| 7 | medical expulsive therapy | 288 | |
| 8 | ((((((urolithiasis) OR ureterolithiasis) OR ureteral calculi) OR nephrolithiasis) OR colic) OR (stone* or calculi* or colic and ureter*)) OR medical expulsive therapy | 121151 | |
| 9 | tamsulosin | 1709 | |
| 10 | silodosin | 356 | |
| 11 | (tamsulosin) OR silodosin | 1938 | |
| 12 | ((((((((urolithiasis) OR ureterolithiasis) OR ureteral calculi) OR nephrolithiasis) OR colic) OR (stone* or calculi* or colic and ureter*)) OR medical expulsive therapy)) AND ((tamsulosin) OR silodosin) | 273 | |
| Recent queries in EMBASE on May 15, 2018 | | | |
| 1 | ((((((((urolithiasis) OR ureterolithiasis) OR ureteral calculi) OR nephrolithiasis) OR colic) OR (stone* or calculi* or colic and ureter*)) OR medical expulsive therapy)) AND ((tamsulosin) OR silodosin) | | 271 |
| Recent queries in Cochrane database on May 15, 2018 | | | |
| 1 | ((((((((urolithiasis) OR ureterolithiasis) OR ureteral calculi) OR nephrolithiasis) OR colic) OR (stone* or calculi* or colic and ureter*)) OR medical expulsive therapy)) AND ((tamsulosin) OR silodosin) | | 255 |
| Recent queries in Scopus on May 12, 2018 | | | |
| 1 | ((((((((urolithiasis) OR ureterolithiasis) OR ureteral calculi) OR nephrolithiasis) OR colic) OR (stone* or calculi* or colic and ureter*)) OR medical expulsive therapy)) AND ((tamsulosin) OR silodosin) | | 196 |
