# Supplementary material for: Silodosin versus tamsulosin for medical expulsive treatment of ureteral stones: A systematic review and meta-analysis
Source: PLoS One. 2018 Aug 28;13(8):e0203035. doi: 10.1371/journal.pone.0203035 (PMC6112672; doi:10.1371/journal.pone.0203035)
Supplement: S2 Table — (DOCX) [file pone.0203035.s003.docx]

S2Table Baseline characteristics of the included studies

| Study | Gender(% of male) | | Stone location (laterality, % of right) | |
| --- | --- | --- | --- | --- |
|  | Silodosin | Tamsulosin | Silodosin | Tamsulosin |
| Gupta 2013 | 36 | 40 | 64 | 54 |
| Rathi 2014 | NA | NA | NA | NA |
| Imperatore 2014 | 50 | 50 | 58 | 46 |
| Kumar 2015 | 71.1 | 69.9 | NA | NA |
| Dell’Atti 2015 | 66.7 | 58.2 | 56.1 | 73.1 |
| Georgescu 2015 | 62 | 54 | 44 | 48 |
| Elgalaly 2016 | 67.3 | 62.7 | 61.5 | 54.9 |
| AS 2016 | 80 | 70 | NA | NA |
| Reddy 2016 | 52 | 56 | 46 | 52 |
| Sharma 2016 | NA | NA | NA | NA |
| Arda 2017 | 69.8 | 62.2 | NA | NA |
| Fahmy 2017 | NA | NA | NA | NA |
| Antony 2017 | NA | NA | NA | NA |
| Rahman 2017 | 55 | 60 | NA | NA |
| Priyanka 2017 | 51.4 | 60 | NA | NA |
| Sentürk 2018 | 54.1 | 50 | NA | NA |

Footnotes: NA, not available;
